# Supplementary material for: Real-life experience with personally familiar faces enhances discrimination based on global information
Source: PeerJ. 2016 Jan 4;4:e1465. doi: 10.7717/peerj.1465 (PMC4741065; doi:10.7717/peerj.1465)
Supplement: Table S1 — These were obtained per condition and group by randomly sampling subjects with replacement; this process was repeated 999 times, leading to a distribution of bootstrapped estimates of the mean accuracy and RT for each condition. Accuracy scores were considered above chance and below ceiling if the confidence intervals did not contain .5 nor 1, which was the case across groups and experimental conditions. [file peerj-04-1465-s001.docx]

**Supplemental material**

Table S1 provides the accuracy scores and correct RTs per condition along with 95% bootstrapped confidence intervals. These were obtained per condition and group by randomly sampling subjects with replacement; this process was repeated 999 times, leading to a distribution of bootstrapped estimates of the mean accuracy and RT for each condition. Accuracy scores were considered above chance and below ceiling if the confidence intervals did not contain .5 nor 1, which was the case across groups and experimental conditions.

Table S1. Average accuracy scores and RTs in msec along with 95% bootstrap confidence intervals for each condition.

| Group | Stimuli | Condition | Accuracy [95% CI] | RTs [95% CI] |
| --- | --- | --- | --- | --- |
| Experimental | Familiar | Full 20% | .62 [.56; .67] | 1056 [939; 1203] |
|  |  | Full 50% | .98 [.96; .99] | 765 [689; 851] |
|  |  | Blur 50% | .89 [.82; .95] | 881 [824; 944] |
|  | Unfamiliar | Full 20% | .65 [.58; .72] | 1062 [952; 1173] |
|  |  | Full 50% | .87 [.83; .91] | 881 [812; 950] |
|  |  | Blur 50% | .66 [.59; .72] | 910 [844; 978] |
| Control | Familiar | Full 20% | .77 [.73; .81] | 994 [878; 1118] |
|  |  | Full 50% | .92 [.89; .95] | 813 [717; 933] |
|  |  | Blur 50% | .79 [.75; .83] | 869 [774; 983] |
|  | Unfamiliar | Full 20% | .77 [.71; .82] | 954 [807; 1126] |
|  |  | Full 50% | .89 [.85; .93] | 883 [747; 1052] |
|  |  | Blur 50% | .70 [.63; .76] | 907 [790; 1033] |
